# Multiple Ossification Centers of the Pubic Bone as a Supportive Radiographic Feature for *COL2A1*-Related Congenital Spondyloepiphyseal Dysplasia

**DOI:** 10.3390/diagnostics16131955

**Published:** 2026-06-23

**Authors:** Vladimir Kenis, Tatiana Markova, Evgeniy Melchenko, Daria Gorodilova

**Affiliations:** 1 H. Turner National Medical Research Center for Children’s Orthopedics and Trauma Surgery, Parkovaya 64–68, Pushkin, 196603 Saint-Petersburg, Russia; 2 Research Centre for Medical Genetics, Moskvorechye St., 1, 115522 Moscow, Russia

**Keywords:** pubic bone, multiple ossification centers, *COL2A1*, skeletal dysplasias, congenital spondyloepiphyseal dysplasia

## Abstract

**Background/Objectives:** During analysis of patients with genetically confirmed *COL2A1*-related skeletal dysplasias, we observed an unusual ossification pattern of the superior pubic ramus characterized by multiple ossification centers—a phenomenon not previously reported in this context. The study aimed to determine the consistency and frequency of multiple ossification centers of the superior pubic ramus in patients with *COL2A1*-related skeletal dysplasias. **Methods:** We retrospectively analyzed pelvic radiographs from 135 patients with genetically confirmed pathogenic *COL2A1* variants. Patients were classified into four clinical subgroups: SEDC, MED, Kniest dysplasia, and Stickler syndrome. **Results:** Multiple ossification centers were identified in 20 (15.5%) of the included patients, all of whom had the SEDC phenotype. The sensitivity of this radiographic sign for SEDC was 36.36%, with high specificity (99.07%) and accuracy (96.5%) compared with other *COL2A1*-related phenotypes and historical general population data. However, these findings require cautious interpretation given the limitations of historical control data. **Conclusions:** We identified an uncommon atypical ossification pattern of the superior pubic ramus that may serve as a supportive radiographic feature when interpreted in conjunction with clinical and genetic findings.

## 1. Introduction

Significant advances in and the increased accessibility of genetic testing have been evident in recent years. Nevertheless, clinical and radiographic evaluation remains essential for diagnosing and managing rare hereditary diseases, including skeletal dysplasias. *COL2A1*-associated skeletal dysplasias represent a substantial proportion of all skeletal dysplasias. Their clinical manifestations form a spectrum, ranging from the mildest, manifesting late in life (early osteoarthritis), to lethal dysplasias characterized by extreme short stature and significant anatomical defects. The clinical spectrum of *COL2A1*-related skeletal dysplasias, according to the 2023 revision of “Nosology of genetic skeletal disorders” includes spondyloepiphyseal dysplasia congenita [1].

Although some genotype–phenotype correlations have been identified, no recurrent variants have been consistently associated with strictly defined clinical forms within this spectrum. Instead, *COL2A1*-related skeletal dysplasias manifest as a continuum of clinical presentations [2].

Congenital spondyloepiphyseal dysplasia (SEDC) is a classical *COL2A1*-related skeletal dysplasia. In typical cases, it manifests with disproportionate short stature, abnormal epiphyses, and flattened vertebral bodies with manifestations presented at birth [3]. Key radiographic manifestations of SEDC in infancy include delayed ossification of the skeleton with absence of the ossification centers of the pubic bones and knee epiphyses, and absent ossification of the vertebral bodies of the upper cervical spine, as well as small, dorsally wedged vertebral bodies of the thoracic and lumbar spine, and delayed ossification of the sacrum. In childhood, radiographic findings include retarded ossification of the pelvis with horizontal acetabular roofs, absent or retarded ossification of the femoral head and neck, coxa vara (occasionally preserved ossification and normal angulation of the femoral neck), varying degrees of epiphyseal and metaphyseal abnormalities of the long tubular bones, and delayed appearance of the carpal and tarsal ossification centers [4].

SEDC is the prototype of the “SEDC family”, the composition of which has expanded and contracted over time with successive publications due to the description of new forms and the reclassification of previously described phenotypes [5].

Radiologic hallmarks of SEDC include delayed endochondral ossification of juxtatruncal bones, delayed ossification of thoracic and lumbar vertebral bodies (with a characteristic pear-shaped appearance), and severely delayed pubic bone ossification that is commonly not appreciated at birth. Epiphyseal ossification is also delayed, and long bones are short and broad, occasionally showing mild dumbbell deformities.

Determining the specific clinical form—not merely the genetic variant—within diseases associated with pathogenic variants in the same gene (particularly *COL2A1*) is important for prognosis, including the prediction of linear growth [6] and complication risk. For instance, SEDC patients have a higher risk of cervical instability and scoliosis, while Stickler syndrome patients are more prone to ophthalmological and auditory disorders. This information is valuable for parents and caregivers to facilitate prevention and early detection [7].

Radiographs of the pelvis, which Gajarajulu et al. called “a window to skeletal dysplasias” [8], are one of the most important sources of diagnostic information [9].

A number of radiographic features of pelvic bones ossification have been described in patients with *COL2A1*-related skeletal dysplasias. In particular, SEDC is characterized by short, broad pelvic wings with horizontal acetabular roofs and narrow sciatic notches [10].

Abnormal, delayed or, depending on the age, absent ossification of the pubic bones is known in SEDC, as well as in cleidocranial dysostosis, achondrogenesis, hypochondrogenesis, Sjogren–Larssen syndrome, Wolf syndrome, and congenital spondyloepiphyseal dysplasia [8,9,11,12,13].

Apart from delayed ossification, other features of superior pubic ramus ossification in SEDC and *COL2A1*-related skeletal dysplasias have not been described. While analyzing data from a large cohort of patients with genetically confirmed *COL2A1*-related skeletal dysplasias, we observed an unusual ossification pattern in several patients with SEDC, namely multiple ossification centers.

This phenomenon has not been previously noted in published studies on *COL2A1*-related skeletal dysplasias. A literature search indicates that this finding is rare and that data on this topic are extremely limited. Extensive studies of pelvic imaging in newborns [14] and children [15] have not reported additional pubic bone ossification centers.

The only publication directly addressing radiographic patterns of pubic bone ossification in children and noting the possibility of multiple ossification centers is the landmark 1956 study by Caffey and Madell [16]. They analyzed pelvic radiographs from 1286 randomly selected newborns, classifying the primary pubic ossification center at birth into three types based on shape and extent. A fourth type encompassed all cases with two or more ossification centers in one or both pubic bones. Multiple ossification centers were identified in 19 of the 1286 infants (1.5%). The most common radiographic manifestation was a vertical radiolucent strip, which in several cases resembled a fracture line. The age of closure varied, occurring as early as 5 months and as late as 1 year.

As Perez-Rossello et al. noted [17], curiously, this distinctive normal variant has received scant mention in the literature. Their study represents the second (after 50 years) and to date the last investigation of this phenomenon, albeit from the perspective of differential diagnosis with child abuse.

Therefore, we hypothesized that this ossification pattern may be more common in patients with *COL2A1*-related skeletal dysplasias. The aim of this study was to determine the consistency and frequency of multiple ossification centers of the superior pubic ramus in patients with *COL2A1*-related skeletal dysplasias, specifically comparing SEDC with other clinical forms.

## 2. Materials and Methods

We conducted a retrospective analysis of pelvic radiographs of 135 patients with various skeletal dysplasias with genetically confirmed pathogenic *COL2A1* variants. The study utilized a database compiled through long-term collaboration by a group of geneticists and orthopedic surgeons from two tertiary academic centers in the Russian Federation. The main clinical and genetic data of this cohort were previously published [18] and subsequently supplemented.

**Figure 1:**
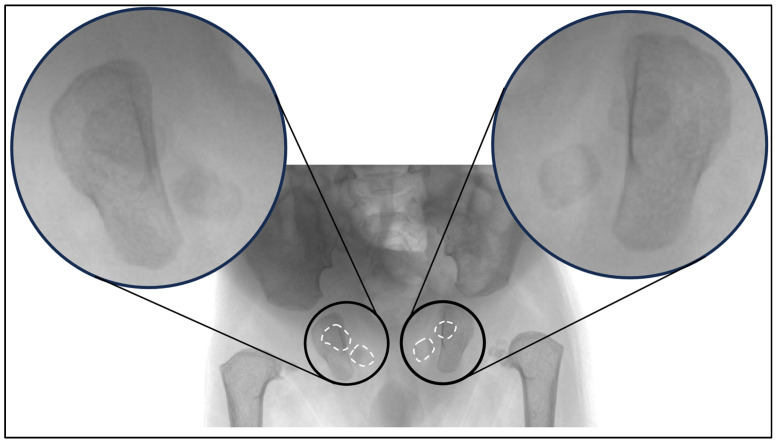
Anteroposterior pelvic radiograph of a 1-year-old child with SEDC showing multiple ossification centers within the superior pubic ramus, visualized as distinct bony parts (white broken lines).

**Figure 2:**
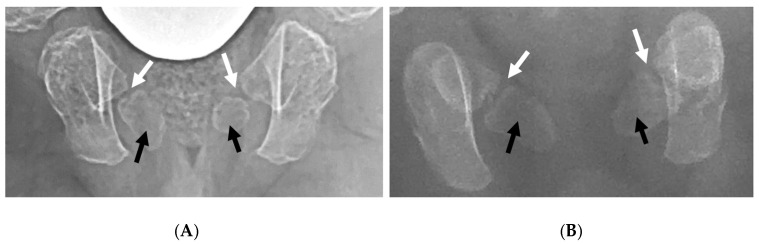
Enlarged anteroposterior pelvic radiographs of 18-month-old (**A**) and 36-month-old (**B**) patients with SEDC demonstrating multiple ossification centers as distinct bony parts (black arrows) separated by a radiolucent strip (white arrows).

**Figure 3:**
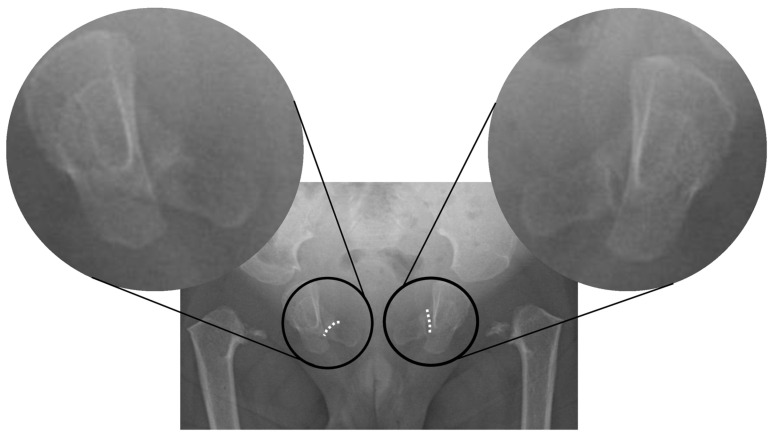
Anteroposterior pelvic radiograph of a 2.5-year-old patient with SEDC showing multiple ossification centers as a vertical radiolucent line dividing the ramus into medial and lateral portions (white broken lines).

**Figure 4:**
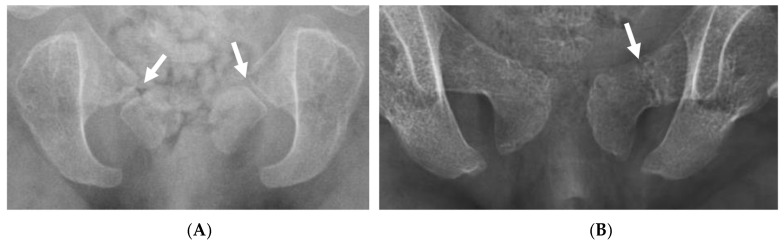
Enlarged anteroposterior pelvic radiographs of 3-year-old (**A**) and 4.5-year-old (**B**) patients with SEDC demonstrating bilateral (**A**) and unilateral (**B**) multiple ossification centers as vertical radiolucent lines dividing the ramus (white arrows).

The inclusion criteria for the study were as follows:

- Non-lethal manifestations of skeletal dysplasia.
- Confirmed pathogenic *COL2A1* variant.
- Availability of at least one anteroposterior pelvic radiograph with symmetrical positioning and clear visualization of both pubic bones.
- Absence of any history of pelvic trauma or surgery.
- Presence of two or more separate ossification centers within the superior pubic ramus, appearing as either distinct bony parts separated by a radiolucent strip (with or without marginal sclerosis) (Figure 1 and Figure 2) or as a vertical radiolucent line dividing the ramus into medial and lateral portions (Figure 3 and Figure 4). Both unilateral and bilateral findings were included.

This definition of multiple ossification centers follows that originally proposed by Caffey and Madell and Perez-Rossello et al. [16,17].

Radiographic evaluation was performed independently by two pediatric orthopedic surgeons (V.K. and E.M.) blinded to patients’ clinical and genetic diagnoses. Each reviewer assessed all available pelvic radiographs for multiple ossification centers of the superior pubic ramus as defined in the inclusion criteria. Discrepancies were recorded, and a third reviewer resolved disagreements when consensus could not be reached. Interobserver agreement was assessed using Cohen’s kappa coefficient with 95% confidence intervals. Assignment of patients to phenotypic groups was based on a combination of clinical and radiographic features provided in the fundamental “*An Atlas of Genetic Disorders of Skeletal Development*” [4]. The main excerpts from this atlas concerning the “classic” phenotypes (SEDC, Kniest dysplasia, Stickler syndrome) and the inclusion criteria for the SED group are presented in Supplement Table S1.

Statistical analysis was performed using StatTech software v. 4.8.0 (StatTech LLC., Kazan, Russia, https://stattech.ru, assessed on 19 June 2026). Sensitivity, specificity, and accuracy were calculated with 95% confidence intervals using the Wilson score method. Positive and negative predictive values were calculated with 95% confidence intervals using the standard formulas. Fisher’s exact test was used to compare the proportion of multiple ossification centers between SEDC and other phenotypic subgroups. A *p*-value < 0.05 was considered statistically significant.

## 3. Results

In our group of 135 patients with *COL2A1*-related skeletal dysplasias, pelvic radiographs were available for 129.

Multiple ossification centers were found in 20 (15.5%) of the included patients. All patients with multiple ossification centers had the SEDC phenotype (Table 1). Among the 20 patients with multiple ossification centers, 14 patients had bilateral involvement and 6 had unilateral involvement (left in 4, right in 2).

Interobserver agreement for the detection of multiple ossification centers was substantial, with Cohen’s kappa = 0.89 (95% CI: 0.78–0.96). Initial agreement between the two independent reviewers was achieved in 121 of 129 cases (93.8%). Disagreements (8 cases) were resolved by consensus discussion with the third reviewer, who was blinded to the initial independent assessments.”

Sensitivity of the sign for SEDC was 36.36% (95% CI: 23.8–50.9%), specificity 99.07% (95% CI: 94.7–99.9%), with accuracy 96.5% (95% CI: 92.4–98.5%). The positive predictive value for SEDC was 95.2% (95% CI: 74.1–99.8%) and the negative predictive value was 75.9% (95% CI: 67.8–82.5%), assuming the prevalence of SEDC within the *COL2A1*-related skeletal dysplasias cohort (40.7%). When comparing the SEDC group to all other phenotypes combined, the difference in the frequency of multiple ossification centers was highly significant (Fisher’s exact test, *p* < 0.0001).

Sensitivity and specificity of this radiographic sign were calculated for patients with *COL2A1*-related skeletal dysplasias and separately for SEDC compared to the general population. Since we did not have our own data on the frequency of this sign in the population, we used published data from Caffey and Madell [16]. For SEDC versus general population, sensitivity was 36.36% (95% CI: 24.2–50.1%), specificity 99.07% (95% CI: 98.4–99.5%) and accuracy 96.5% (95% CI: 94.1–98.1%).

We also analyzed the occurrence of the trait by age (Table 2).

As the data presented in Table 2 show, multiple ossification centers were observed with equal frequency in children under 1 year of age and from 1 to 10 years of age. After 10 years, we did not observe this sign.

Among the 20 patients with multiple ossification centers, genetic variants included glycine substitutions in the triple helix domain (*n* = 17, 85%), non-glycine substitutions (*n* = 1, 5%), and in-frame deletions (*n* = 2, 10%). This distribution was comparable to the overall SEDC cohort. No patient with multiple ossification centers had variants outside the triple helix domain, although the small sample size precludes meaningful statistical comparison.

## 4. Discussion

Molecular genetic analysis is currently the standard for diagnosing rare skeletal diseases. However, despite its high accuracy and increasing availability, clinical and radiographic diagnostics remain the first line of investigation [19,20]. With increasing knowledge of hereditary skeletal diseases, it has become clear that patients with this group of pathologies have virtually no pathognomonic clinical or radiographic features. Most of these features, although with varying frequencies, are clearly detectable in other diseases and even physiological conditions.

Abnormal ossification and formation of the pubic bones can be occasionally found as a developmental abnormality [21,22,23].

Therefore, defining additional diagnostic criteria enables increased diagnostic vigilance, narrowing the scope of genetic testing, and overall optimizing the diagnostic process.

Chondrification centers in the pubic bones are well developed by seven to eight weeks. The pubic ossification centers appear between five and six intrauterine months. Ossification initiates in the superior pubic ramus, anterior to the acetabulum. In the early stages of ossification the pubis is dumbbell-shaped, and is the smallest and most delicate of the pelvic elements [24].

Primary ossification centers of the pelvis initiate in utero, followed postnatally by secondary ossification at a range of locations, and these processes not complete until adulthood. This cascade of events can vary between individuals [24,25].

Postnatal ossification of the pelvis is well documented with a focus on the differential diagnosis with trauma and assessment of skeletal maturity [26,27,28].

The unusual pubic bone ossification pattern observed in children with SEDC demonstrated high specificity both relative to the general population (based on published data) and compared with patients who had other *COL2A1*-related skeletal dysplasias.

Numerous attempts have been made to establish clear genotype–phenotype relationships in *COL2A1*-related skeletal dysplasias. However, a strong, reliable prediction of phenotype has not been achieved. Notably, when describing these types, authors often use vague phrases such as ‘often associated with…’ or ‘often variants in…’ [1]. Despite the obvious patterns associated with the complex structure of the *COL2A1* protein, there is clear variability, making phenotype prediction largely conditional. Nevertheless, a number of consistent phenotypic groups within this spectrum are evident. At the time w)hen classical descriptions of skeletal dysplasias were formulated, genetic confirmation was lacking, and clinicians and radiologists had to rely on careful observation. However, some less noticeable features may well have escaped attention.

For complex pathologies such as *COL2A1*-related skeletal dysplasias, Handa et al. proposed two principles for categorizing clinical findings [29]. First, one can categorize a particular case into a specific skeletal dysplasia family according to recognition of the overall radiologic pattern; second, one can make the diagnosis on the basis of subtle radiological findings. The first step, pattern recognition, in particular is the key to diagnosing skeletal dysplasias correctly.

The phenotypic classification of *COL2A1*-related skeletal dysplasias remains debated. Historically, distinct clinical syndromes were described, and later genetic analysis confirmed *COL2A1* as the causative gene. Several fundamental sources of *COL2A1* classification can be found in the literature. According to “Nosology of genetic skeletal disorders”, the following variants can be distinguished: *COL2A1*-related achondrogenesis, hypochondrogenesis, platyspondylic dysplasia, spondyloepiphyseal dysplasia congenita (SEDC), *COL2A1*-related spondyloepimetaphyseal dysplasia, *COL2A1*-related SED with metatarsal shortening, Kniest dysplasia, spondyloperipheral dysplasia, *COL2A1*-related Stickler syndrome, *COL2A1*-related dysplasia of the proximal femoral epiphyses [1]. Another well-known source of information on the phenotypes of hereditary diseases is OMIM. It distinguishes 16 phenotypes, some of which overlap with the previous source https://www.omim.org/entry/120140?search=COL2A1 (accessed on 19 June 2026). Recently, several fundamental studies have proposed their own versions of the classification of *COL2A1* phenotypes and genotypes, based on clinical, radiological, and genetic principles [10,18,29]. However, clear genotype–phenotype correlations have not been established [2].

In our previous study [18], we identified 4 phenotype groups based on a combination of clinical and radiographic features. For this study, we also used this pragmatic classification into 4 phenotypes. The decision to assign a particular patient to one of the 4 groups was made by expert consensus among the authors (see “Section 2”).

This radiographic phenomenon—specific ossification of the superior pubic ramus—represents a small but potentially valuable component of the SEDC radiographic phenotype. It complements the overall diagnostic picture and may contribute to a better understanding of skeletal formation processes in health and disease.

Beyond our observation of this atypical ossification pattern, the literature emphasizes the need for differential diagnosis with child abuse [30,31]. Perez-Rossello et al. analyzed clinical, imaging, and outcome data from 14 cases (age range 1.5 to 9 months) with abnormal radiographic findings of the superior pubic ramus. In four cases (29%), the findings were classified as normal variants; in three (21%), as fractures; and in seven (50%), as indeterminate. Although SEDC is rare, its coincidence with child abuse is even less likely, though not impossible, particularly given the increasing participation of children with disabilities in sports and other activities [30,31].

Comprehensive analysis of previously published radiographs from children with *COL2A1*-related skeletal dysplasias is challenging, primarily due to the poor quality and unsystematized presentation of images. In later publications, however, we identified at least one clear case: a patient with SEMD Strudwick type and the p.Gly1122Arg mutation had multiple ossification centers of both superior pubic rami at age 6.8 years, although this was not noted in Terhal et al.’s article [10]. A more detailed analysis of published data may reveal additional cases, but this was not the objective of this study.

Notably, no published studies have observed multiple ossification centers in children older than one year. In our cohort, however, 10 of the 20 patients with multiple centers were between one and ten years of age. This finding suggests that the presence of multiple ossification centers beyond one year of age may have high specificity for SEDC, a finding that warrants validation in larger, controlled studies.

Our study has several limitations. First, radiographic data were collected unsystematically: pelvic radiographs were obtained at different ages and using different devices, and analysis was retrospective. Nevertheless, the clarity of the radiographic feature enabled detection in most cases. Sensitivity and specificity were assessed relative to the general population using literature data. Although this phenomenon has not been previously described in the radiographic diagnosis of *COL2A1*-related skeletal dysplasias, retrospective analysis of databases at skeletal dysplasia referral centers may help clarify its prevalence and quantify sensitivity and specificity.

We categorized patients with *COL2A1*-related skeletal dysplasias into four groups. Because the diagnostic criteria for these groups are not entirely clear, experts (orthopedic surgeons and medical geneticists with extensive experience) reached consensus on group assignment.

In this study, we did not correlate the observed phenomenon with the patients’ genotypes to avoid an excessive number of variables, as the primary objective of this article was to describe a novel radiographic feature and assess its diagnostic utility rather than to establish genotype–phenotype relationships. As previously noted, the data were obtained through the analysis of a large database of patients with SEDC. In a subsequent systematic analysis of this database, we plan to conduct a genotype–phenotype correlation for this feature. This represents an important direction for future research.

The observation that multiple ossification centers were not observed in any patient older than 10 years warrants further consideration. This finding suggests that the atypical ossification pattern represents a temporary developmental phenomenon rather than a persistent anatomical variant. In normal skeletal development, the pubic bone typically ossifies from a single primary center that appears during late fetal life and expands progressively. The presence of multiple centers in SEDC patients may reflect a more fundamental disturbance of endochondral ossification—specifically, a delay or fragmentation of the normal coalescence process. However, we cannot exclude sampling bias: older patients in our cohort had fewer radiographs available, and the retrospective design may have missed cases where the sign persisted but was not imaged. Prospective longitudinal imaging would be required to confirm the age-dependent disappearance.

Collagen type II is the major structural component of the cartilaginous anlagen of endochondrally forming bones. Pathogenic variants in *COL2A1* disrupt the normal extracellular matrix structure, potentially interfering with the signaling pathways that coordinate chondrocyte differentiation and ossification center formation. The fact that multiple centers can persist until age 10 years in SEDC patients—whereas in the general population they typically fuse by 5–12 months—suggests a profound delay in the maturation of the pubic symphysis region. After age 10 years, the ossification centers ultimately fuse, indicating that the fusion process, although delayed, eventually occurs. This pattern parallels other known developmental delays in SEDC, including delayed appearance of the femoral head ossification centers and delayed carpal bone maturation.

The biological mechanisms underlying this phenomenon remain speculative. One possibility is that the abnormal collagen matrix creates physical barriers to the diffusion of ossification-promoting factors or disrupts the cell–cell communication required for coordinated ossification. Alternatively, the mechanical environment of the pubic ramus—which undergoes significant changes with weight-bearing and ambulation—may influence ossification center fusion, and the delayed motor development typical of SEDC patients (who often walk later than healthy peers) could contribute to the prolonged persistence of multiple centers. Future studies using animal models of *COL2A1* mutations may help elucidate these mechanisms.

We acknowledge several important limitations regarding the use of historical control data from Caffey and Madell [16]. The imaging quality and techniques used in the mid-20th century differ substantially from modern radiographic methods, potentially affecting the detection rate of small ossification centers. The population characteristics (including nutritional status, prenatal care, and demographic factors) may differ from our contemporary Russian cohort. Radiographic interpretation standards have evolved over the past seven decades, and current observers may have different thresholds for identifying subtle radiolucencies. These factors could potentially bias our specificity estimates. However, we note that Caffey and Madell’s study remains the only large-scale systematic assessment of pubic ossification patterns in the general pediatric population; no contemporary study of comparable size has been published. Given current ethical considerations, comparable contemporary data are unlikely to become available, making this unique historical dataset the only available reference. Additionally, given the control data is from newborns while the patient group is in the 0–18 age range, the direct conclusion is methodologically problematic. The unusual ossification pattern of the pubic bone observed in children with SEDC formally demonstrated high specificity in our cohort both for the general population (based on published historical data) and in comparison with patients who had other *COL2A1*-related skeletal dysplasias. However, given the limitations of using historical control data, these specificity estimates should be interpreted cautiously. Consequently, the specificity values presented should be considered as preliminary estimates requiring validation in contemporary age-matched control populations.

## 5. Conclusions

We identified a rare phenomenon of atypical ossification of the superior ramus of the pubic bone, which may represent a supportive radiographic feature in the evaluation of patients with *COL2A1*-related skeletal dysplasias. This finding may be useful in the differential diagnosis of child abuse. Given the retrospective nature of this study and the limited size of non-SEDC subgroups, these findings should be validated in larger, prospective, age-matched cohorts. Further study of this condition may contribute to a better understanding of endochondral skeletal formation and the role of *COL2A1* in this process.

## Figures and Tables

**Table 1:** Frequency of multiple ossification centers of the superior pubic ramus in different *COL2A1*-related phenotypes and in the general population (historical data from Caffey & Madell, 1956 [16]).

	SEDC	Kniest Dysplasia	Stickler Syndrome	SED	General Population (Caffey and Madell, 1956 [16])
Total	55	12	32	30	1286
Multiple ossification centers	20	0	0	0	19
One ossification center	31	12	32	30	1266
No ossification	4	0	0	0	1

**Table 2:** Ossification patterns of the superior pubic ramus in SEDC patients by age group.

	Age		
Ossification	Up to 1 Year	1–10 Years	>10 Years
Multiple ossification centers	10	10	0
One ossification center	8	14	7
No ossification	4	0	0
Total	24	24	7

## Data Availability

The data presented in this study are available on request from the corresponding author due to privacy.

## Supplementary Materials

The following supporting information can be downloaded at: https://www.mdpi.com/article/10.3390/diagnostics16131955/s1, Table S1: Major diagnostic findings.

## Abbreviations

The following abbreviations are used in this manuscript:

SEDC	Congenital spondyloepiphyseal dysplasia
SED	Spondyloepiphyseal dysplasia
SEMD	Spondyloepimetaphyseal dysplasia
*COL2A1*	Collagen type II alpha 1 chain
