# Supplementary material for: Multiple Ossification Centers of the Pubic Bone as a Supportive Radiographic Feature for COL2A1-Related Congenital Spondyloepiphyseal Dysplasia
Source: Diagnostics (Basel). 2026 Jun 23;16(13):1955. doi: 10.3390/diagnostics16131955 (PMC13359935; doi:10.3390/diagnostics16131955)
Supplement: Supplementary file 1 [file diagnostics-16-01955-s001.zip › diagnostics-4328507-supplementary.pdf]

Phenotype	Major clinical findings	Major radiographic features	
		infancy	childhood
SEDC*	Disproportionate dwarfism with short spine, short neck<br>Normal-sized hands and feet in most cases.<br>Flat face<br>Myopia in approximately 50% of cases.<br>Occasionally cleft palate and/or clubfeet.	Retarded ossification of the skeleton with absence of the ossification centers of the pubic bones and knee epiphyses.<br>Absent ossification of the vertebral bodies of the upper cervical spine; small, dorsally wedged vertebral bodies of the thoracic and lumbar spine; regarded ossification of the sacrum.	Flatness, immaturity, and anterior ossification defects of the vertebral bodies.<br>Hypoplasia and delayed ossification of the odontoid process of C-2.<br>Retarded ossification of the pelvis with horizontal acetabular roofs. Absent or retarded ossification of the femoral head and neck. Coxa vara in many patients; occasionally preserved ossification and normal angulation of the femoral neck.<br>Varying degrees of epiphyseal and metaphyseal abnormalities of the long tubular bones. Shortness is proportionate to the severity of epiphyseal and metaphyseal changes.<br>4. Delayed appearance of the carpal and tarsal ossification centers; otherwise relatively normal hands and feet in most patients.
SEMD*	Disproportionate dwarfism with short spine, short neck, progressive scoliosis, pectus carinatum, genu valgum.<br>Normal-sized hands and feet in most cases.<br>Flat face.<br>Myopia in approximately 50% of cases.<br>Occasionally cleft palate and/or clubfeet.	Retarded ossification of the skeleton with absence of the ossification centers of the pubic bones and knee epiphyses.<br>Absent ossification of the vertebral bodies of the upper cervical spine; small, dorsally wedged vertebral bodies of the thoracic and lumbar spine; regarded	Flatness, immaturity, and anterior ossification defects of the vertebral bodies.<br>Hypoplasia and delayed ossification of the odontoid process of C-2.<br>Retarded ossification of the pelvis with horizontal acetabular roofs. Absent or retarded ossification of the femoral head and neck. Coxa vara with marked metaphyseal irregularities.<br>Epiphyseal and metaphyseal abnormalities of the long tubular bones. Shortness and limb deformities are proportionate to the severity of metaphyseal changes.<br>Delayed appearance of the carpal and tarsal ossification centers; otherwise relatively normal short tubular bones.

		ossification of the sacrum.	
Stickler syndrome*	Midface hypoplasia, often becoming less conspicuous with advancing age.<br>Cleft palate in approximately 25% of patients, Pierre Robin sequence in infants.<br>Congenital, often nonprogressive high-degree myopia in most patients; abnormal vitreous with ensuing complications.<br>Sensorineural hearing loss.<br>Conductive hearing loss, often in association with cleft palate and recurrent otitis.<br>Joint hypermobility. In striking cases, prominent joints, joint pain, and morning stiffness.<br>Mild shortness of stature, occasionally normal or increased height with Marfanoid habitus.	Coronal clefts of the vertebral bodies.<br>Widened ends of femora and tibiae.	Mild to moderate flatness of the vertebral bodies, occasionally with irregularities of the upper and lower end plates.<br>Undermodeling of the long tubular bones with broad epiphyses and metaphyses, most notably of the femora and tibiae.
Kniest dysplasia	Peculiar face with flat mid-face and depressed nasal bridge; sometimes shallow orbits with protuberant eyes.<br>Short trunk with dorsal kyphosis, accentuated lumbar lordosis, and sometimes thoracic scoliosis in the later course of the disease.<br>Short and broad thorax with sternal protrusion.<br>Short extremities with prominent joints and restricted joint mobility.<br>Cleft palate in about 50% of cases; frequent myopia, often conductive and neural hearing loss; sometimes club feet.	Platyspondyly with anterior wedging of the vertebral bodies. In infancy frequently coronal cleft of the lumbar bodies.<br>Broad ilia with hypoplasia of basilar portions.<br>Very broad and short femoral necks. The capital femoral epiphyses ossify late, first appearing in the second or third year of life in mild cases and not until adolescence in severe cases.<br>Ultimately, they are large and flattened.<br>Short tubular bones with broad metaphyses and large and deformed epiphyses in the child and adult.	
SED**	Normal birth height<br>Normal or mildly short stature<br>Absent or mild extra-skeletal symptoms<br>Early osteoarthritis	Perthes-like changes<br>Early osteoarthritis<br>Absent spinal changes or mild platyspondyly<br>Mild epiphyseal flattening	

*- excerpted from Spranger JW, Brill PW, Hall C, Nishimura G, Superti-Furga A, Unger S Bone Dysplasias, An Atlas of Genetic Disorders of Skeletal Development, 4th edition, New York, 2018.

**- authors' data (Markova T, Kenis V, Melchenko E, Osipova D, Nagornova T, Orlova A, Zakharova E, Dadali E, Kutsev S. Clinical and Genetic Characteristics of COL2A1-Associated Skeletal Dysplasias in 60 Russian Patients : Part I. Genes (Basel). 2022 Jan 13;13(1):137. doi : 10.3390/genes13010137. PMID: 35052477; PMCID: PMC8775336.)
